# RNA isolation from micro-quantity of articular cartilage for quantitative gene expression by microarray analysis

**DOI:** 10.7150/ijms.65343

**Published:** 2022-01-01

**Authors:** Xiaowei Zhang, Trevor J. McFarland, Kristina Vartanian, Yong Zhu, Christina A. Harrington, Cong-Qiu Chu

**Affiliations:** 1 Division of Arthritis and Rheumatic Diseases, Oregon Health & Science University, Portland, Oregon 97239.; 2 Section of Rheumatology, VA Portland Health Care System, Portland, Oregon 97239.; 3 Gene Profiling Shared Resource, Oregon Health & Science University; Portland, Oregon 97239.; 4 Vivoscript, Inc, P. O. Box 63025, Irvine, CA 92602.; 5 Department of Molecular and Medical Genetics, Oregon Health & Science University; Portland, Oregon 97239.

**Keywords:** RNA isolation, articular cartilage, cartilage repair, microarray

## Abstract

Isolation of quality RNA from articular cartilage has been challenging due to low cellularity and the high abundance of extracellular matrix and proteoglycan proteins. Recently developed methods for isolation of high quality RNA from cartilage are more applicable to larger cartilage specimens typically weighing at least 25 mg. While these methods generate RNA suitable for analysis, they are less successful with smaller tissue inputs. For the study of small focal defect cartilage specimens an improved RNA extraction method is needed. Here we report a protocol for direct RNA isolation from less than 3 mg of wet weight rabbit articular cartilage for quantitative microarray gene profiling. This protocol is useful for identifying differentially expressed genes in chondrocytes following focal cartilage repair and can potentially be adopted for gene expression analysis of cartilage biopsy specimens from human joints.

## Introduction

SRY-type high-mobility group box 9 (SOX9) is a master transcription factor of chondrogenesis. In previous studies we have shown that an engineered cell-permeable super-positively charged SOX9 (scSOX9) protein was able to improve the quality of repaired cartilage by microfracture 1, 2. Microfracture is a common procedure for cartilage repair, but often generates only fibrocartilage, which is inferior to hyaline cartilage in function and durability. In a rabbit cartilage injury model, applied at the site of microfracture, scSOX9 successfully helped regenerate hyaline-like cartilage. We are interested in identifying gene expression profiles to elucidate the metabolism of cartilage regeneration and cartilage defect repair.

Isolation of intact RNA from adult articular cartilage is challenging. Cartilage is characterized by low cellularity and a high content of highly cross-linked extracellular matrix proteins with aggregating proteoglycans 3. Both classes of molecules are extremely large and highly negatively charged. Direct isolation of RNA from articular cartilage is historically problematic with low yield, low purity, and poor integrity of the extracted RNA. Most gene expression studies are based on RNA extracted from cultured chondrocytes. *In situ* hybridization can represent an alternative technique to examine gene expressions in cartilage, but it is not quantitative 4. Several developed protocols are capable of successfully isolating quality RNA from articular cartilage but require large amounts of tissue 5-8*.* Zheng et al reported that good quality RNA can be isolated from 25 mg of tissue but this required pooling eight femoral heads of young mice (2-3 weeks of age) 8. However, cartilage specimens harvested from individual repaired cartilage sites result in small quantities of tissue and hence these published protocols for RNA isolation are not suitable. Thus, our main goal was to develop a reliable protocol for the isolation of quality RNA for quantitative gene expression analysis from micro-quantities of tissue harvested from repaired cartilage at the site of the focal defect. With our modified method, we successfully isolated RNA from less than 3 mg of cartilage (wet weight) of sufficient quality for gene expression analysis.

## Materials and Methods

### Cartilage tissue

All animal studies were approved by Institutional Animal Care and Use Committee of the VAPORHCS, #3469-15). Mature female New Zealand white rabbits with body weights of 3.0 - 3.5 kg were used. A cylindrical osteochondral defect of 4 mm in diameter and 3 mm in depth is made at the patella groove of the femur. This is followed by microfracture 1 supplemented with collagen membrane or collagen membrane carrying scSOX9 or a loss-of-function mutant, scSOX9-A76E. Eight weeks following the procedure, full thickness cartilage plugs of 4 mm in diameter were harvested from the repaired cartilage in the defect area. All cartilage samples were weighed immediately upon harvest (weights ranged from 1.9 to 2.9 mg) and either snap frozen in liquid nitrogen or stored in 350 µL of TRIzol (Thermo Fisher Scientific). All samples were stored at -80°C until the RNA isolation step was performed.

### RNA isolation

**Figure 1:**
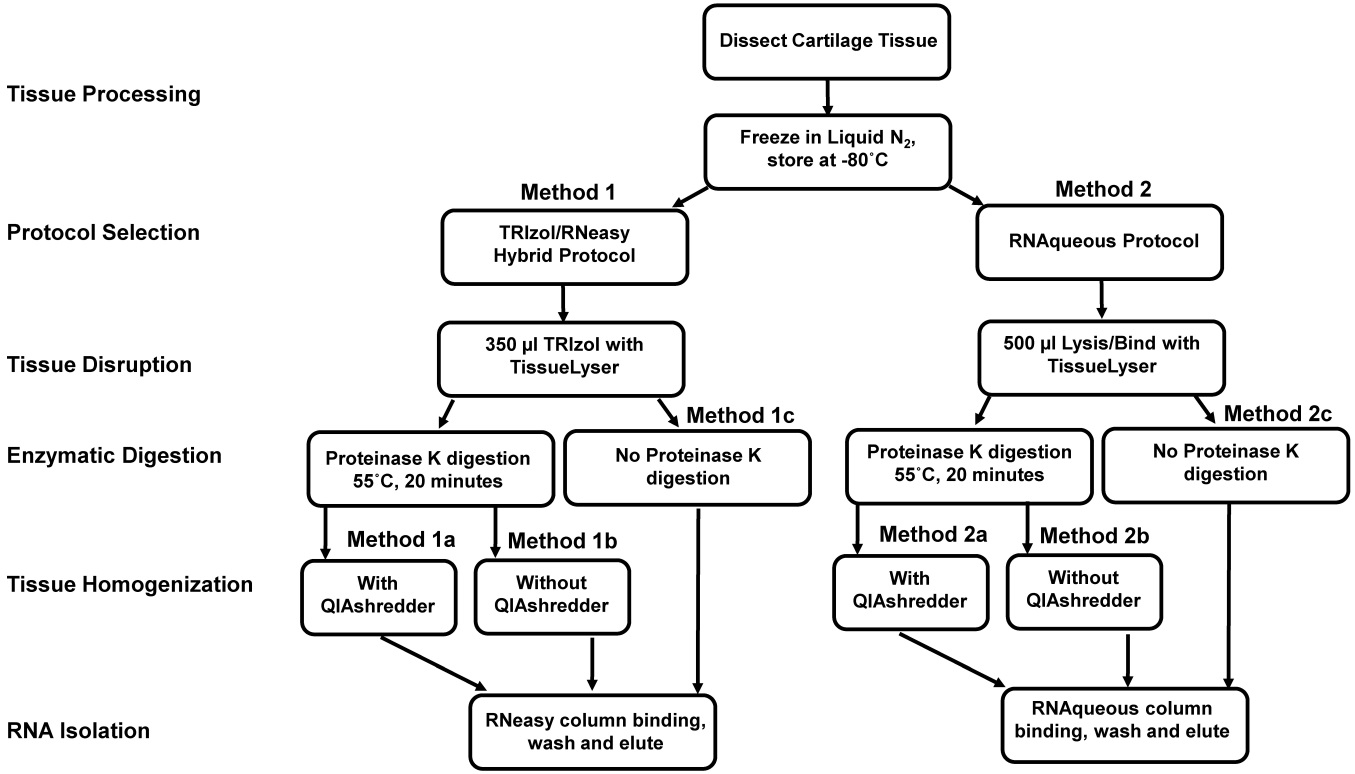
Flow chart of protocols for RNA isolation from articular cartilage. Two methods with different workflow modifications were tested for isolation of quality RNA from articular cartilage for cDNA microarray assay for gene expression. Normal rabbit cartilage tissue was collected from distal femoral surface of knee joint and either snap-frozen or suspended in 350 µl and stored at -80^o^C. The detailed protocols are described in Table 1.

RNA isolation and microarray assays were performed at the Oregon Health & Science University Gene Profiling Shared Resource. Quality control of RNA isolation was performed according to the quality assessment procedures developed by the core laboratory 9. To improve the RNA isolation process from this challenging tissue source two methods with various modifications were evaluated (see flow chart in Figure 1 and Table 1 for detailed methodology): 1. A modified TRIzol (Thermo Fisher Scientific) extraction followed by an RNeasy column purification (QIAGEN) 10 or 2. A modified RNAqueous (Ambion) extraction. Samples stored in TRIzol were thawed and disrupted using stainless steel beads and a TissueLyser II device (QIAGEN) set at 50Hz for 5 minutes. Samples that had been flash frozen were thawed after the addition of 500 µL of RNAqueous lysis buffer; these samples were also disrupted with the TissueLyser II as above. RNA was isolated per manufacturer's instructions with the following modifications: each protocol was tested with and without the addition of a Proteinase K (QIAGEN) digestion step using 10 µl of the enzyme (>600mAU, incubated at 55°C for 20 minutes) and with and without a viscosity-reducing homogenization step using a QIAshredder column (QIAGEN). Following the various tissue pre-processing steps, the lysates were passed through either an RNeasy (QIAGEN) or RNAqueous (Ambion) RNA binding column and washed with kit specific buffers. RNA was eluted with 30-50 µL of nuclease-free water (Figure 1 and Table 1). RNA concentration, yield, and purity ratios were measured by UV absorbance using a Nanodrop 1000 Spectrophotometer (ThermoFisher Scientific). RNA size distribution and integrity were determined using a 2100 Bioanalyzer (Agilent Technologies) and an RNA 6000 Pico chip. For samples assayed on the Bioanalyzer with the Total RNA Program, Agilent software uses an algorithm to assign an RNA integrity score based on the trace pattern. This score is a value between 1 and 10 and is referred to as an RNA integrity number (RIN) 11, 12. Based on initial results obtained using the RNAqueous method, this procedure was tested on only a few samples due to the limited number of samples available. Ultimately, based on overall RNA quality and yield results (Table 2), Method 1a, the TRIzol method with Proteinase K digestion followed by QIAshredder column homogenization and RNeasy column purification (QIAGEN) (Figure 1 and Table 1), was selected for preparing samples for downstream expression profiling.

### Microarray analysis

The input for microarray target preparation was 30 ng of total RNA. RNA was reverse-transcribed, amplified and labeled with biotin using the Ovation Pico WTA v.2 RNA amplification kit (NuGEN Technologies) with the Encore Biotin Labeling Module. Amplified target cDNA yield was determined by OD_260_ absorbance and all samples passed vendor QC thresholds. Amplified and labeled cDNA targets were combined with hybridization solution and controls (ThermoFisher Scientific, formerly Affymetrix). Three micrograms of labeled target in 130 µl of hybridization solution was injected into a GeneChip Rabbit Gene 1.0 array cartridge (ThermoFisher Scientific, formerly Affymetrix) designed to interrogate over 20,000 rabbit genes. Arrays were incubated for 18 hours at 45 °C and processed as per manufacturer's instructions. Arrays were scanned using the GeneChip Scanner 3000 7G with autoloader. Image processing was performed using the Affymetrix GeneChip Command Console (AGCC) software. Each array file was then analyzed using Transcriptome Analysis Console v4.0.2 software (ThermoFisher Scientific) for differential gene expression.

## Results

### RNA quantity and quality

Yield and quality of RNA extracted from approximately 2-3 mg of rabbit cartilage using the different methods described is shown in Table 2. Based on RNA yield and Bioanalyzer RIN scores Method 1a performed best among all the methods tested and generated good quality RNA from normal cartilage. Therefore, subsequent RNA isolation for all the samples from repaired cartilage was carried out using Method 1a (Figure 1 and Table 1) which includes a Proteinase K treatment and QIAshredder homogenization.

Table 3 summarizes the quality and purity of RNA isolated from samples at 8 weeks post microfracture supplemented with scSOX9 or controls. Adequate yields of RNA for microarray assay were isolated from individual samples ranging in wet weight from 1.9 to 2.9 mg. Concentrations measured by UV absorbance were between 3.5 - 87.4 ng/µl and were generally consistent with Bioanalyzer estimated concentrations. The optical density (OD) 260/280 ratios ranged from 1.7- 2.0, indicating acceptable RNA purity for microarray assay.

**Figure 2:**
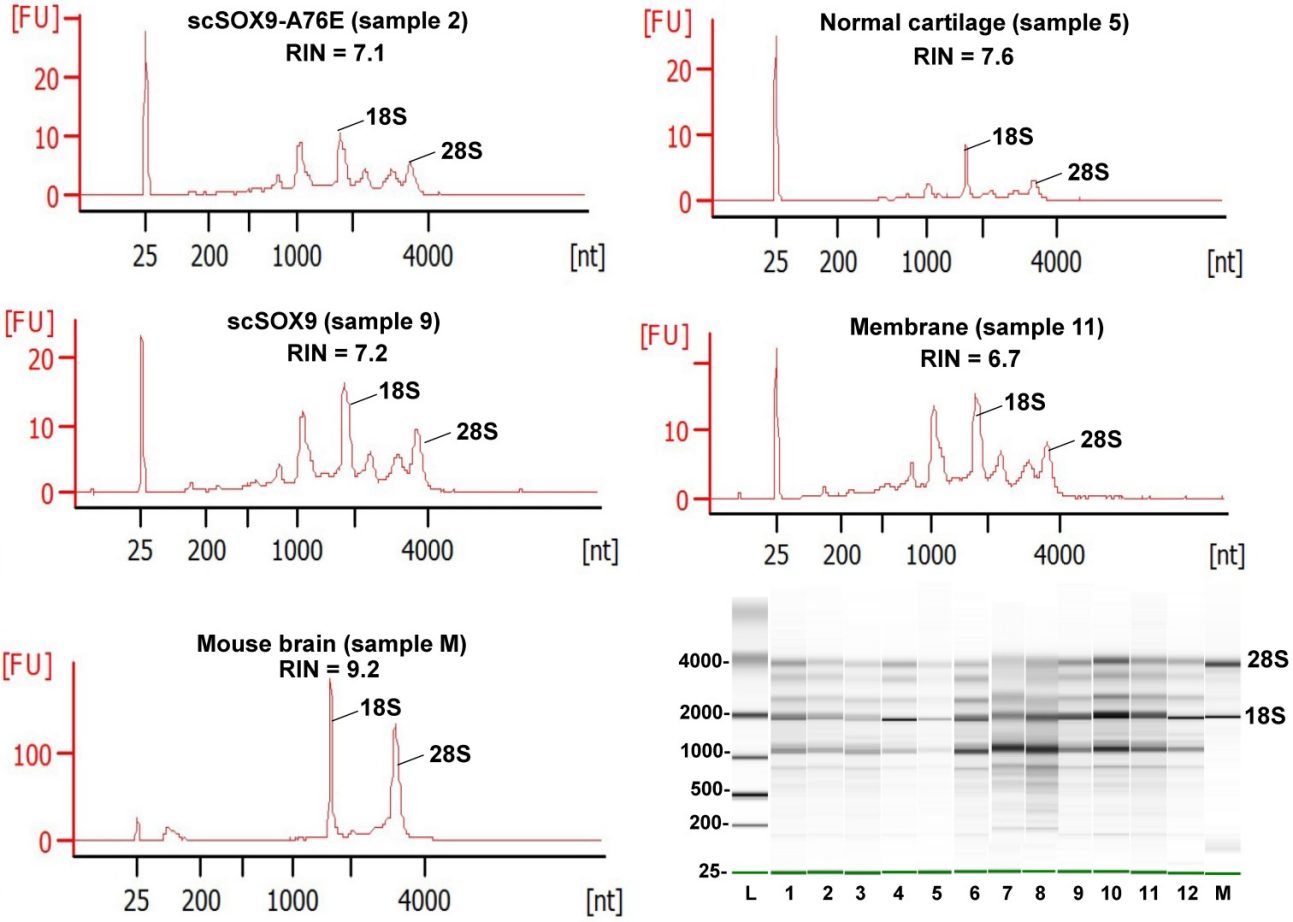
Quality assessment of RNA extracted from repaired cartilage. Bioanalyzer traces of RNA of selected samples from normal cartilage, collagen membrane only (Membrane), scSOX9 or scSOX9-A76E treated cartilage and electrophoresis graphs are shown. L: RNA ladder; normal cartilage (sample 5, 7, 8); Membrane only (sample 1, 11, 12); scSOX9 (sample 4, 9, 10); scSOX9-A76E (sample 2, 3, 6). M: RNA isolated from mouse brain is shown for comparison.

The Bioanalyzer electropherogram traces of all samples displayed discrete ribosomal RNA peaks at 18S and 28S with several additional peaks between the 18S and 28S bands and just below the 18S band (Figure 2). RIN scores ranged from 6.5 - 8.3, indicating the extracted RNA was largely intact.

### Quality assessment of microarray analysis

cDNA target yields prior to array hybridization were 9.4 ±1.552 µg (mean ± SD, n = 12) demonstrating that all RNA samples were capable of generating expected yields of cDNA for hybridization. Assessment of the sample hybridization performance metrics generated with Affymetrix GeneChip Command Console (AGCC) processing of array data indicated that RNA input quality was good and array hybridizations performed well according to GeneChip standard quality thresholds (9). Positive and negative Area Under the Curve (AUC) 13 measures how well the probe set signals separate the positive controls from the negative controls and is a useful indicator of overall array performance with each sample. AUC values of our samples were 0.89 ± 0.004 (mean ± SD, n = 12). AUC values approaching 0.9 and above are considered indicative of good sample performance 14.

### Differential gene expression

**Figure 3:**
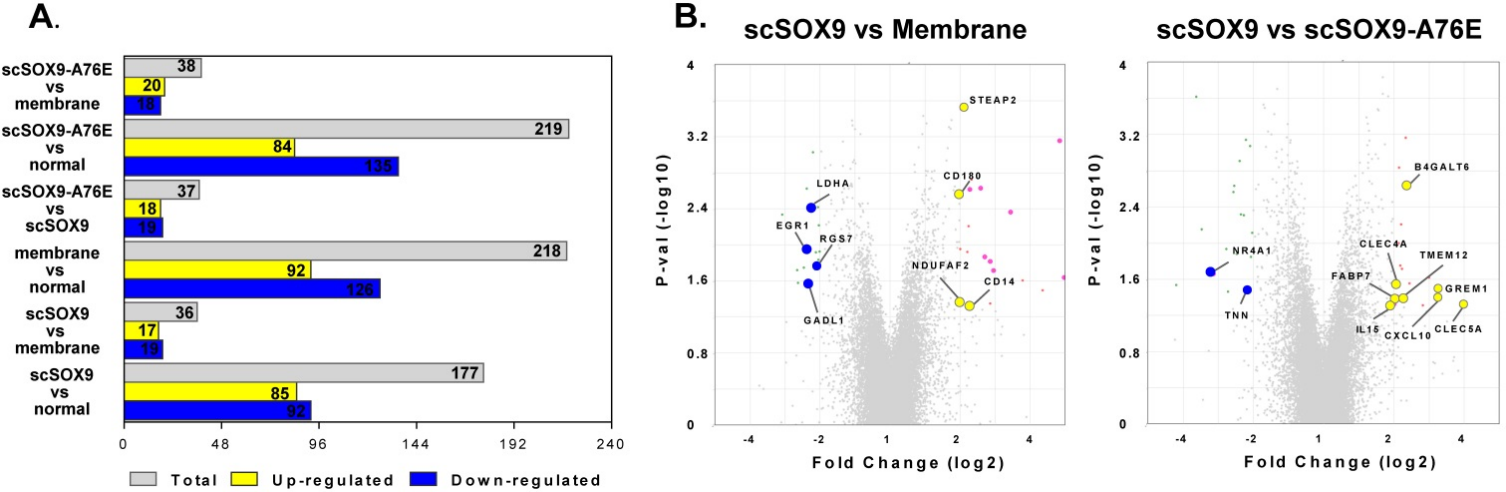
Changes of gene expression at 8 weeks post microfracture. Microarray assay detected differently expressed genes during cartilage repair induced by scSOX9 supplemented microfracture. (A) Total number and up-regulated and down-regulated genes with changes of ≤ - 2 or ≥ 2 folds (p < 0.05) comparing different treatment. (B) Volcano plots with highlight of changes of genes induced scSOX9 compared with collagen membrane only or with scSOX9-A76E.

The purpose of our study was to compare the gene expression profiles of cartilage regeneration induced by scSOX9 compared with mutant scSOX9-A76E and collagen membrane only and in reference to normal cartilage. The scSOX9 treated samples resulted in a total of 177 significantly differentially expressed genes (≤ -2 or ≥ 2 fold change, p < 0.05) compared with normal cartilage; 36 genes compared with membrane only and 37 genes compared with loss-of-function mutant scSOX9-A76E (Figure 3). Interestingly, the most prominently upregulated genes in scSOX9 vs. scSOX9-A96E were related to extracellular matrix homeostasis such as Beta-1,4-Galactosyltransferase 6 (B4GALT6) and C-type lectin domain family 4 member A (CLEC4A), bone differentiation such as gremlin 1, DAN family BMP antagonist (GREM1) and C-type lectin domain containing 5A (CLEC5A), and inflammation (interleukin-15 and CXCL10). More analyses of differential gene expression in specimens of repaired cartilage at 4, 8 and 12 weeks post microfracture are under way.

## Discussion

Direct isolation of sufficient amounts of high quality RNA from articular cartilage has been challenging largely due to the inherit nature of cartilage. Typically large amounts of cartilage tissue are required to achieve good RNA yields 15, 16. Highly cross-linked proteoglycans present in cartilage can also interfere with RNA purity. This has been circumvented by extracting RNA from isolated chondrocytes derived from cartilage 17. A recently published method successfully purified RNA with an average RIN value of 7.9 ± 03 from cartilage of a human osteoarthritis specimen. The quality of RNA isolated using this method met the requirement for RNA sequencing but required a 100 mg of cartilage 15. Others have reported methods for isolation of quality RNA from cartilage with starting material as low as 25 mg in quantity from young cartilage 8. Gehrsitz et al reported that 10 - 40 mg cartilage plugs obtained from human autopsy specimen was sufficient for isolation of quality RNA for RT-PCR 18. Though high quality RNA is directly extracted from cartilage tissue, none of these protocols suits the requirement of our cartilage specimens due to the small size of the focal injury area. Our method took advantage of commercially available RNA isolation kits with specific modifications that yielded a sufficient amount of quality RNA for microarray analysis using micro quantities of cartilage tissue. In particular, the addition of Proteinase K digestion followed by QIAshredder homogenization yielded a high quantity of RNA with good quality. The RIN of RNA isolated from this method ranged from 6.5 - 8.3 and in 50% of the samples, an RIN score of at least 7 (as recommended for RNA sequencing) was achieved 19. As evidenced by these results, it is our belief that complete digestion and homogenization is crucial for the release of RNA from this highly cross-linked protein-rich tissue source. This method is particularly suitable for RNA extraction from articular cartilage in osteoarthritis or cartilage injury and repair models with small animals where a large quantity of specimen is not available. This method may also be adopted for RNA isolation from cartilage biopsy specimens in human osteoarthritis studies.

SOX9 is the master transcription factor required for chondrogenesis and orchestrates the expression of many genes during this process 20. However, much of our knowledge about SOX9 action during chondrogenesis is learnt from *in vitro* studies on cultured chondrocytes 20 and may not fully represent *in vivo* mechanisms. Using the recombinant scSOX9 in combination with microfracture, we have shown that scSOX9 is able to induce hyaline-like cartilage regeneration in a rabbit acute cartilage injury model 1. This model offered a unique opportunity to understand the interaction of SOX9 with other molecules *in vivo* during cartilage repair by inducing bone marrow derived mesenchymal stem cells (MSCs). Since the target genes for scSOX9 during the process of cartilage repair are largely unknown, we employed microarray analysis for gene expression which allows simultaneous examination of over 20,000 genes 21. The use of microarray technology to study thousands of genes involved in cartilage regeneration has not been reported, although RNA-sequencing has been recently used to study isolated chondrocytes 22, 23. We, therefore, made an effort to modify methods to isolate quality RNA from a micro amount of cartilage specimen to satisfy the requirement for microarray assay. In our preliminary analysis, scSOX9 induced cartilage regeneration was associated with genes involved in cell proliferation and differentiation, inflammation, energy metabolism, cell survival, and extracellular matrix protein homeostasis.

Limitations of our study must be noted. First, good quality RNA as judged by RIN was isolated from micro quantity of cartilage by our modified method. Although it is widely used for assessment of total RNA integrity, RIN does not necessarily reflect the integrity of mRNA 12. For example, inconsistencies between RIN and corresponding RNA electropherogram profiles and lack of correlation of RIN with RNA concentration were observed in postmortem human brain tissue were observed 24. Second, bulk RNA isolation followed by microarray analysis cannot precisely assign the differentially expressed genes to specific cell types. Single cell RNA-sequencing may be helpful in achieving that end and is being considered for future studies. Third, the cartilage samples obtained from repaired cartilage tissue may not represent a pure chondrocyte cell type but may also contain MSCs which are in transition status. Forth, using collagen membrane to carry scSOX9 in our experimental model may not be the ultimate method to apply for human cartilage repair; and collagen membrane might have impact on scSOX9 induced gene expression.

## Figures and Tables

**Table 1:** RNA isolation methodology.

Method 1: Modified Trizol/RNeasy Hybrid	
1.	Disrupt tissue with a 5 mm stainless steel bead using a TissueLyzer II instrument (Qiagen) in 350 µL of TRIzol at 50Hz for 5 minutes. Store the homogenate at room temperature for 5 minutes.
2.	Pass lysate through QIAshredder viscosity-reducing homogenization column (Qiagen) (Step tested during protocol refinement, see Figure 1).
3.	Add 10 µL of Proteinase K (Qiagen) and incubate at 55°C for 20 minutes (Step tested during protocol refinement, see Figure 1).
4.	Add chloroform to the homogenate (0.2 mL chloroform per 1 mL TRIzol) and shake vigorously for 20 seconds, then allow the sample to sit at room temperature for 2-3 minutes.
5.	Spin at 10,000 g for 18 minutes at 4˚C.
6.	Carefully remove aqueous phase (top) by aspiration and transfer to new sterile RNase-free tube (1.5 ml tube).
7.	Slowly add an equal volume of 100% ethanol, mix as needed.
8.	Load the sample (up to 700 µL) into an RNeasy column (Qiagen kit) seated in a collection tube and spin for 30 seconds at 8,000 g. Discard flow-through. Repeat as necessary.
9.	Add 700 µL buffer RW1 onto column and spin 30 seconds at 8,000 g. Discard flow-through.
10.	Transfer column into a new collection tube, add 500 µL buffer RPE and spin for 30 seconds at 8,000 x g. Discard flow-through. Ensure ethanol has been added to the RPE buffer before use.
11.	Add 500 μL buffer RPE and spin 2 minutes at 8,000 g. Discard flow-through.
12.	Spin the column for 1 minute at 8,000 g to get rid of any residual buffer in the column.
13.	Transfer the column to a new 1.5 ml collection tube and pipet 30-50 µL of RNase-free water directly onto the column membrane. Allow the sample to sit at room temperature for 1 - 2 minutes, and then spin 1 minute at 8,000 x g to elute RNA.
14.	Store RNA at -80°C until use.
**Method 2: Modified RNAqueous**	
1.	Disrupt tissue with a 5 mm stainless steel bead using a TissueLyzer II instrument (Qiagen) in 500 µL of RNAqueous lysis buffer at 50 Hz for 5 minutes. Store the homogenate at room temperature for 5 minutes.
2.	Pass lysate through QIAshredder viscosity-reducing homogenization column (Qiagen) (Step tested during protocol refinement, see Figure 1).
3.	Add 10 µL of Proteinase K (Qiagen) and incubate at 55°C for 20 minutes (Step tested during protocol refinement, see Figure 1).
4.	Add equal volume of 64% ethanol.
5.	Load the sample into an RNAqueous column supplied with the kit.
6.	Wash column with 700 µL of Wash buffer #1.
7.	Wash column with 2 x 500 µL Wash buffer #2/3.
8.	Elute RNA with 40 µL pre-heated (75⁰C) elution solution.
9.	Store RNA at -80°C until use.

**Table 2:** Comparison of yield and quality of RNA isolated from rabbit cartilage using modified methodologies*

RNA Quality Comparison	Method 1a^†^	Method 1b	Method 1c	Method 2a	Method 2b	Method 2c
Method Details	Trizol/RNeasy Hybrid with Proteinase K and QIAshredder	Trizol/RNeasy Hybrid with Proteinase K	Trizol/RNeasy Hybrid without Proteinase K	Modified RNaqueous with Proteinase K and QIAshredder	Modified RNaqueous with Proteinase K	Modified RNaqueous without Proteinase K
Number of samples tested	**24**	**9**	**1**	**1**	**2**	**2**
UV absorbance 260/280 (average)	**1.9**	**1.8**	**2.9**	**2.2**	**0.0^‡^**	**5.2^‡^**
UV absorbance 260/230 (average)	**1.2**	**0.9**	**1.8**	**1.0**	**0.0^‡^**	**0.0^‡^**
Bioanalyzer Concentration (ng/μl) (average)	**58**	**38**	**8.1**	**0.8**	**0.0**	**0.1**
Bioanalyzer Total Yield (μg) (average)	**1.7**	**1.1**	**0.3**	**0.03**	**0.0**	**0.0**
Bioanalyzer RIN Score (average)	**7.1**	**5.2**	**Not applicable**	**2.2**	**Not applicable**	**Not applicable**
Representative Bioanalyzer Trace	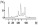	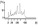	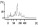	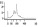	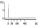	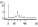

*Normal rabbit articular cartilage (3 mg per sample) was used for RNA isolation in each method; ^†^Method chosen for microarray analysis; **^‡^**UV data available for only one sample.

**Table 3:** Quantity and quality of RNA isolated from cartilage samples harvested at 8 weeks post microfracture*

Project samples^†^		NanoDrop One			BioAnalyzer (Pico & Nano chip assay)		
**Sample ID**	**Treatment^‡^**	**260/280**	**RNA concentration (ng/µl)**	**RNA yield (µg)**	** RIN**	**Estimated RNA concentration (ng/µl)**	**RNA yield (µg)**
1	Membrane	2.0	55.3	1.7	7.0	52.8	1.6
2	scSOX9-A76E	1.9	47.6	1.4	7.1	33	1.0
3	scSOX9-A76E	1.7	63.8	1.9	6.5	35.2	1.1
4	scSOX9	1.8	9.8	0.3	6.9	12.4	0.4
5	Normal	2.0	22.0	0.7	7.6	13.2	0.4
6	scSOX9-A76E	2.0	42.7	1.3	8.3	62.4	1.9
7	Normal	1.9	3.5	0.1	7.5	2.2	0.1
8	Normal	2.0	8.2	0.2	6.8	10.1	0.3
9	scSOX9	2.0	66.4	2.0	7.2	57.6	1.7
10	scSOX9	2.0	87.4	2.6	6.5	140.8	4.2
11	Membrane	2.0	45.7	1.4	6.7	59.2	1.8
12	Membrane	1.9	60.1	1.8	6.7	54.4	1.6

*Data presented in this table were obtained for RNA isolated with Method 1a (Figure 1 and Table 1). **^†^**Wet weight of cartilage samples ranged between 1.9 - 2.9 mg. **^‡^**Treatment: After cartilage defect was created, microfracture was performed and supplemented with collagen membrane (Membrane) alone, or with collagen membrane carrying super-positive changed charged SRY-type high-mobility group box 9 (scSOX9) or a mutant scSOX9-A76E. Normal: normal cartilage was taken from the counter lateral knee. RIN: RNA integrity number.
